# Factors associated with alexithymia among the Lebanese population: results of a cross-sectional study

**DOI:** 10.1186/s40359-019-0353-5

**Published:** 2019-12-11

**Authors:** Sahar Obeid, Marwan Akel, Chadia Haddad, Kassandra Fares, Hala Sacre, Pascale Salameh, Souheil Hallit

**Affiliations:** 1 Psychiatric Hospital of the Cross, P.O. Box 60096, Jall-Eddib, Lebanon; 2 Faculty of Arts and Science, Holy Spirit University of Kaslik (USEK), Jounieh, Lebanon; 3 INSPECT-LB: Institut National de Sante Publique, Epidemiologie Clinique et Toxicologie, Beirut, Lebanon; 4 School of Pharmacy, Lebanese International University, Beirut, Lebanon; 5 Drug Information Center, Order of Pharmacists of Lebanon, Beirut, Lebanon; 6 Faculty of Pharmacy, Lebanese University, Beirut, Lebanon; 7 Faculty of Medicine, Lebanese University, Beirut, Lebanon; 8 Faculty of Medicine and Medical Sciences, Holy Spirit University of Kaslik (USEK), Jounieh, Lebanon

**Keywords:** Alexithymia, Stress, Burnout, Anxiety

## Abstract

**Background:**

To our knowledge, no research project on alexithymia has been conducted in Lebanon. The objective of this study was to assess risk factors associated with alexithymia in a representative sample of the Lebanese population.

**Methods:**

This is a cross-sectional study, conducted between November 2017 and March 2018, which enrolled 789 participants from al districts of Lebanon. The Toronto Alexithymia Scale (TAS-20) was used to measure alexithymia, the Alcohol Use Disorders Identification Test to assess alcohol use, drinking patterns, and alcohol-related issues, the Rosenberg self-esteem scale to evaluate self-worth, the Hamilton depression rating scale and Hamilton Anxiety Scale to screen for depression and anxiety respectively, the Three-Dimensional Work Fatigue Inventory to measure physical, mental and emotional work fatigue respectively, the Columbia–Suicide Severity Rating Scale to evaluate suicidal ideation and behavior, the Perceived Stress Scale to measure stress, the Liebowitz Social Anxiety Scale to help identify a social anxiety disorder and the Quick Emotional Intelligence Self-Assessment to measure emotional intelligence.

**Results:**

The results showed that 395 (50.4%) were not alexithymic, 226 (28.8%) were possible alexithymic, whereas 163 (20.8%) were alexithymic according to established clinical cutoffs. Stress (Beta = 0.456), emotional exhaustion (Beta = 0.249), the AUDIT score (Beta = 0.225) and anxiety (Beta = 0.096) were associated with higher alexithymia, whereas low emotional work fatigue (Beta = −0.114) and being married (Beta = −1.933) were associated with lower alexithymia.

People in distress (Beta = 7.33) was associated with higher alexithymia scores, whereas people with high wellbeing (Beta = −2.18), an intermediate (Beta = −2.90) and a high (Beta = −2.71) family monthly income were associated with lower alexithymia compared to a low one.

**Conclusion:**

Alexithymia appears to be influenced by many factors, including stress, anxiety, and burnout. To reduce its prevalence, it is important that health professionals educate the public about these factors. Further studies on a larger scale are needed to confirm our findings.

## Background

Alexithymia is “*a personality construct that refers to one’s inability to successfully deal with emotional regulation*” [1]. This cross-cultural observable fact recognized in studies across 18 different ethnic and racial groups [2], was coined by Sifneos who describes it as a deflection of emotions [3]. Alexithymia is characterized by a difficulty identifying one’s feelings and describing them to others, limited imaginal manners and a stimulus-bound, externally oriented cognitive style [4]. Alexithymic people have difficulties in regulating their emotions. The low emotion regulation level is associated with low levels of social ability, emotion expression and emotion intelligence [5]. Moreover, alexithymic persons have impaired ability to understand their own feelings and those of others [6]. Alexithymia was originally reported to be widespread in psychosomatic patients who have trouble in developing satisfactory interactions with therapists and in adhering to psychological and behavioral programs. Soon after, these traits were found in other neuropsychiatric diseases such as substance use disorder, posttraumatic stress disorder, panic disorder, and somatoform pain disorder [7, 8].

Numbers have shown that alexithymia is a personality character widely present in a population [9]: using the Toronto Alexithymia Scale (TAS-20) cutoff scores, its prevalence have been reported at 10.0% in the German population [10] and 12.8% in the Finnish population [11]. Among the working age population, the prevalence of alexithymia ranged between 9 and 17% for men and 5–10% for women [11].

Alexithymia has been shown to be associated with socio-demographic factors such as gender, advanced age, low educational level and low socioeconomic status [ 11–13], and mental health problems including [14] 1) somatoform disorders 2) alcohol use disorder because alcohol may offer a coping strategy to boost interpersonal performance in individuals uncomfortable in a social setting [15] 3) substance use disorder 4) work-related burnout [16, 17] and perceived stress, which is defined as a psychological state or process through which individuals perceive threat to their physical and psychological well-being 5) depression [18–20] and anxiety [21, 22] 6) social phobia [23], and 7) eating disorders [24–28]. Consequently, alexithymia may be a coping or defense strategy to challenging situations [29].

In addition to aforementioned risk factors, a negative association was found between struggle in expressing emotions and self-rated self-esteem [30]. Moreover, studies [31, 32] showed that alexithymia and emotional intelligence are not related but are robustly inversely correlated constructs: the existence of alexithymic traits in individuals is a sign of low emotional intelligence. In fact, highly alexithymic persons have difficulty using their emotions to guide their behavior, a reduced stress tolerance, and inadequate adaptive resources [33].

The main benefit of cluster analysis is that similar participants can be grouped together. This helps identify patterns, reveal associations, and outline structure between participants. The emergence of a clear structure out of this analysis can allow easier decision-making.

Based on the alexithymia theory, higher alexithymia is more likely to be seen in people with negative emotions [34]. Since 2012, the big number of Syrian refugees (more than a million) that came to Lebanon had a negative its impact on the economy, politics and society [35, 36]; the Lebanese civil war had many negative consequences on the mental health, as mental disorders were seen in about one third of the Lebanese population [37]. However, mental disorders remain underreported as Lebanese do not often seek the help of a specialist to diagnose and treat mental symptoms due to cultural norms [37]. Finally, and to our knowledge, no research project on alexithymia has been conducted in Lebanon. Therefore, the objective of the present study was to assess factors (alcohol dependence, self-esteem, depression, anxiety, stress, social anxiety, emotional intelligence, suicidal ideation and behavior, work fatigue) and different clusters associated with alexithymia in a sample of the Lebanese adult population.

## Methods

Between November 2017 and March 2018, 789 community dwelling participants were enrolled from all Lebanese governorates/regions, using a proportionate random sample. Each governorate is divided into Caza, which is divided into multiple villages. Two villages were randomly chosen, from which participants were randomly selected. Adults (>18 years old) were eligible to participate. Excluded were those who refused to fill the questionnaire, and those who self-reported psychiatric problems (such as schizophrenia, bipolar disorder, drug abuse), mental retardation and dementia, which would make it difficult to understand and complete the study questionnaire. Trained clinical psychologists performed data collection through personal interviews with the participants. They had a training prior to launching data collection to ensure the quality of research and avoid interrater variability as much as possible. A clinical psychologist, independent of this study, also clinically evaluated the level of psychiatric illness in the study group to exclude those with psychiatric problems. The same methodology was used in previous papers [38–47].

### Minimal sample size calculation

According to a population size of 6,000,000 in Lebanon, a prevalence of 24.6% of alexithymic subjects based on a Jordanian study [48] (in the absence of similar local studies), and a 95% confidence level, the minimal sample size needed was 285 according to the Epi info software.

### Questionnaire

The questionnaire used was in Arabic, the native language of Lebanon. The first part assessed sociodemographic characteristics of the included participants (age, gender, education level, marital status, socioeconomic level, type of alcohol drunk), and the other part consisted of the different scales used in this study:

#### Toronto alexithymia scale (TAS-20)

This 20-items scale [49] was used to assess alexithymia. Items are rated using 5-point Likert scale from 1 = strongly disagree to 5 = strongly agree. The cut-off scoring of TAS-20 is: ≤ 51 = non-alexithymia, 52–60 = possible alexithymia, and ≥ 61 = alexithymia. The TAS-20 has acceptable validity and reliability [50, 51].

#### The alcohol use disorders identification test (AUDIT)

The self-reported ten-item scale was used to assess alcohol use [52]. Alcohol consumption was considered dangerous when participants scored 8 or more.

#### Rosenberg self-esteem scale (RSES)

This 10-item scale evaluates self-worth by measuring both positive and negative feelings about oneself [53]. Answers were graded from 1 (strongly agree) to 4 (strongly disagree), with higher scores indicating higher self-esteem.

#### Hamilton depression rating scale (HDRS)

The validated Arabic version of the HDRS was used in this study [54] [55], with higher scores reflecting higher depression.

#### Hamilton anxiety scale (HAM-A)

The HAM-A [56], recently validated in Lebanon [57], consists of 14 items, rated from 0 (symptoms not present) to 4 (very severe symptoms); higher scores reflect higher anxiety.

#### The three-dimensional work fatigue inventory (3D-WFI)

It consists of a total of 18 questions (3 packs of 6 questions each) and measures physical, mental and emotional work fatigue respectively [58]. Item scoring ranged from 0 = never to 4 = every day. Higher scores indicate higher fatigue in all 3 dimensions.

#### Columbia-suicide severity rating scale (C-SSRS)

This six-item instrument evaluates suicidal ideation and behavior, with a score of 0 indicating the absence of suicidal ideation, whereas a score of 1 or more reflects its presence [59].

#### The perceived stress scale (PSS)

This ten-item instrument is used to evaluate stress in the last month, with answers graded from 0 (never) to 4 (very often); higher scores reflect higher perceived stress.

#### Liebowitz social anxiety scale (LSAS)

This self-reported scale contains 13 questions relate to performance anxiety and 11 to social situations [60], with higher scores reflecting higher social fear and avoidance [61].

#### The quick emotional intelligence self-assessment

Four subscales, each composed of 10 questions, derive from this scale: emotional awareness, emotional management, social emotional awareness and relationship management. Items are measured from 0 (never) to 4 (always), with higher scores reflecting higher emotional intelligence for all subscales [62].

All scales were translated from English to Arabic through an initial translation and a back translation process. A mental health specialist translated the English version into Arabic, and then this version was translated back into English by another specialist. Upon completion of this process, translators compared the English versions of all scales to determine if the variables had the same meaning. The Cronbach’s alpha values were calculated for all the scales as follows: TAS (0.778), AUDIT (0.885), RSES (0.733), HDRS (0.890), HAM-A (0.898), physical work fatigue (0.823), mental work fatigue (0.667), emotional work fatigue (0.909), C-SSRS (0.762), PSS (0.667), LSAS total score (0.954), LSAS fear subscale (0.945), LSAS avoidance subscale (0.953), emotional awareness (0.823), emotional management (0.888), social emotional awareness (0.902) and relationship management (0.908).

### Statistical analyses

Data analysis was conducted using SPSS software version 23. The independent-sample t-test was used when comparing two means. For categorical variables, the Chi-2 was used when applicable. A stepwise linear regression was conducted taking the alexithymia score as the dependent variable and taking all variables that showed a *p* < 0.1 in the bivariate analysis as independent variables. Moreover, Cronbach’s alpha was recorded for reliability analysis for all the scales. A *P*-value less than 0.05 was considered significant.

Patterns among specific samples can be concluded from the factor and cluster analyses. An exploratory factor analysis was conducted as a first step to classify patterns of the different factors associated with alexithymia in the current sample, with the extraction being done via a promax rotation. The results of the Kaiser–Meyer–Olkin (KMO) index and Bartlett’s Chi-square test of sphericity ensured the adequacy of the sample. Factors with an Eigenvalue higher than one were retained. Items with factor loading >0.4 were considered as belonging to a factor. Afterwards, a cluster analysis was performed using the results of the factor analysis and using the K-mean method to identify the participants’ patterns. The latter method allowed the grouping of the participants into a three-cluster structure, which reflects their profiles.

## Results

A sensitivity analysis (data not shown) was performed for all participants interviewed by different psychologists, to check for discrepancies in the results: none was detected. Thus, the results were considered as one set for all participants.

Of 950 questionnaires distributed, 789 (83.05%) were completed and collected back. The mean age of the participants was 30.30 ± 12.52 years (54.8% males). Other participants’ characteristics can be found in Table 1. According to established clinical cutoffs of the TAS-20, results showed that 395 (50.4%) were not alexithymic, 226 (28.8%) were possible alexithymic, and 163 (20.8%) were alexithymic.

**Table 1:** Sociodemographic characteristics of the sample population

		Frequency (%)
Gender	Male	423 (54.8%)
	Female	349 (45.2%)
Education level	Illiterate	12 (1.6%)
	Primary	39 (5.3%)
	Complementary	52 (7.0%)
	Secondary	113 (15.2%)
	University	462 (62.3%)
	Higher education	64 (8.6%)
Socioeconomic status	<1000 $	376 (50.7%)
	1000–2000 $	260 (35.1%)
	>2000 $	105 (14.2%)
Marital status	Single	488 (63.1%)
	Married	236 (30.5%)
	Widowed	19 (2.5%)
	Divorced	30 (3.9%)
		Mean ± SD
Age (in years)		30.30 ± 12.52

### Factor analysis

Out of all the items in the questionnaire, all variables could be extracted from the list, except for the Liebowitz total score (low communality of 0.284), which was taken out of the factor analysis. The factor analysis for all the scales total score was run over the whole sample (Total = 789). The total items converged over a solution of 3 factors (Factor 1 = High emotional intelligence & low emotional work fatigue; Factor 2 = High physical and mental work fatigue & high stress; Factor 3 = Low self-esteem, high suicidal ideation and alcohol dependence), explaining a total of 66.33% of the variance (KMO = 0.832; Bartlett’s test of sphericity *p* < 0.001) (Table 2).

**Table 2:** Pattern loading of the major factor solutions after promax rotation, taking alexithymia among these factors

	Factor 1	Factor 2	Factor 3
High social emotional awareness	0.875		
High relationship management	0.871		
High emotional management	0.827		
High emotional awareness	0.771		
Low emotional work fatigue	0.759		
High mental work fatigue		0.883	
High perceived stress		0.720	
High physical work fatigue		0.703	
Low self-esteem			0.700
High suicidal ideation			0.647
High alcohol dependence			0.547

Factor 1 = High emotional intelligence & low emotional work fatigue; Factor 2 = High physical and mental work fatigue & high stress; Factor 3 = low self-esteem, high suicidal ideation and alcohol dependence

### Profiles of participants

A cluster analysis based on the three factors, derived three mutually exclusive clusters representing 28.89, 38.65 and 30.67% of all participants, respectively. The first cluster represented people with depersonalization (low emotional intelligence and high emotional work fatigue but low physical and mental work fatigue and low stress), the second represented people with high wellbeing (high emotional intelligence and low emotional work fatigue, with high self-esteem, low suicidal ideation and low alcohol dependence), and the third, people in distress (low self-esteem, high suicidal ideation and high alcohol dependence, with high physical and mental work fatigue and high stress) (Table 3).

**Table 3:** Classification of participants in the study sample by cluster analysis using the categories factor scoring

	Cluster 1 *N* = 228 (28.89%)	Cluster 2 *N* = 305 (38.65%)	Cluster 3 *N* = 242 (30.67%)
Factor 1: High emotional intelligence & low emotional work fatigue	− 0.93	0.91	− 0.28
Factor 2: High physical and mental work fatigue & high stress	−0.71	− 0.10	0.81
Factor 3: Low self-esteem, high suicidal ideation and alcohol dependence	−0.32	− 0.57	1.08

Factor 1 = High emotional intelligence & low emotional work fatigue; Factor 2 = High physical and mental work fatigue & high stress; Factor 3 = Low self-esteem, high suicidal ideation and alcohol dependence

cluster 1 = People with depersonalization (low emotional intelligence and high emotional work fatigue but low physical and mental work fatigue and low stress); cluster 2 = People with high wellbeing (high emotional intelligence and low emotional work fatigue, with high self-esteem, low suicidal ideation and low alcohol dependence); cluster 3 = People in distress (low self-esteem, high suicidal ideation and high alcohol dependence, with high physical and mental work fatigue and high stress)

### Bivariate analysis

A significantly higher mean alexithymia score was found in persons with low familial monthly income (53.49) compared to intermediate (50.78) and high (51.54), and among divorced persons compared to single, married or widowed. In addition, higher alexithymia was significantly and positively correlated with more alcohol dependence (AUDIT score) (*r* = 0.306), more depression (HAM-D score) (*r* = 0.255) and anxiety (HAM-A score) (*r* = 0.367), perceived stress (PSC score) (*r* = 0.433), social phobia (Liebowitz social anxiety scale) (*r* = 0.145), mental work fatigue (*r* = 0.436), higher emotional work fatigue (*r* = 0.175) and higher suicidal ideation (*r* = 0.119). However, less alexithymia score was correlated with higher emotional management (*r* = −0.167), social emotional awareness (*r* = −0.101), relationship management (*r* = −0.142) and higher number of kids (*r* = −0.076) (Table 4).

**Table 4:** Bivariate analysis of the factors associated with the alexithymia score

		TAS score	*p*-value
		Mean ± SD	
Familial monthly income	< 1000 $	53.49 ± 10.30	0.005
	1000–2000 $	50.78 ± 10.65	
	> 2000 $	51.54 ± 10.66	
Marital status	Single	52.57 ± 10.38	0.001
	Married	50.45 ± 10.19	
	Widowed	52.83 ± 9.67	
	Divorced	58.27 ± 11.98	
		Correlation coefficient	*p*-value
Audit score		0.306	<0.001
HAM-D score		0.255	<0.001
HAM-A score		0.367	<0.001
PSC score		0.433	<0.001
Liebowitz social anxiety scale		0.145	<0.001
Emotional awareness		−0.060	0.097
Emotional management		− 0.167	<0.001
Social Emotional awareness		−0.101	0.005
Relationship management		−0.142	<0.001
Mental work fatigue		0.436	<0.001
Physical work fatigue		0.227	<0.001
Emotional work fatigue		0.175	<0.001
Suicidal ideation score		0.119	0.001
Number of kids		−0.076	0.044

### Multivariable analysis

The results of a first linear regression, taking the alexithymia score as the dependent variable, showed that higher alexithymia scores were associated with higher stress (Beta = 0.456), higher mental work fatigue (Beta = 0.249), higher alcohol use disorder (higher AUDIT scores) (Beta = 0.225), higher emotional work fatigue (Beta = 0.114) and higher anxiety (Beta = 0.096), whereas being married (Beta = −1.933) was associated with lower alexithymia scores.

A second linear regression, taking the alexithymia score as the dependent variable and the factors obtained in the factor analysis as independent variables, showed that Factor 2 (High physical and mental work fatigue & high stress) and Factor 3 (Low self-esteem, high suicidal ideation and alcohol dependence) were associated with higher alexithymia (Beta = 0.16 and Beta = 0.19) respectively, whereas Factor 1 (High emotional intelligence & low emotional work fatigue) (Beta = −0.03) was associated with lower alexithymia.

A third linear regression, taking the alexithymia score as the dependent variable and the clusters obtained as independent variables, showed that participants in cluster 3 (People in distress) (Beta = 7.33) had higher alexithymia scores, whereas those in cluster 2 (People with high wellbeing) (Beta = −2.18), an intermediate (Beta = −2.90) and a high (Beta = −2.71) socioeconomic levels had lower alexithymia (Table 5).

**Table 5:** Multivariable analysis

Model 1: Linear regression taking the TAS score as dependent variable and all the scales as independent variables.					
	Unstandardized Beta	Standardized Beta	*p*-value	Confidence interval	
				Lower Bound	Upper Bound
Stress	0.456	0.266	<0.001	0.317	0.596
Mental work fatigue	0.249	0.246	<0.001	0.164	0.333
Emotional work fatigue	0.114	−0.093	0.015	0.022	0.206
Alcohol dependence	0.225	0.170	<0.001	0.126	0.323
Married status	−1.933	−0.082	0.019	−3.552	−0.313
Anxiety	.096	0.088	0.035	0.007	0.184
Model 2: Linear regression taking the TAS score as dependent variable and three factors obtained in the factor analysis as independent variables.					
	Unstandardized Beta	Standardized Beta	*p*-value	Confidence interval	
				Lower Bound	Upper Bound
Factor 2: High physical and mental work fatigue & high stress	0.162	0.339	<0.001	0.130	0.195
Factor 3: Low self-esteem, high suicidal ideation and alcohol dependence	0.197	0.172	<0.001	0.118	0.277
Factor 1: High emotional intelligence & low emotional work fatigue	−0.036	−0.122	<0.001	−0.055	−0.017
Intermediate familial monthly income^a^	−2.745	−0.123	<0.001	−4.267	−1.223
High familial monthly income^a^	−2.875	−0.092	0.008	−5.014	−0.736
Model 3: Linear regression taking the TAS score as dependent variable and all the scales as independent variables.					
	Unstandardized Beta	Standardized Beta	p-value	Confidence interval	
				Lower Bound	Upper Bound
Cluster 3^b^	7.336	0.320	<0.001	5.490	9.182
Intermediate familial monthly income^a^	−2.906	−0.130	<0.001	−4.486	−1.326
Cluster 2^b^	−2.184	−0.103	0.012	−3.893	−0.475
High familial monthly income^a^	−2.712	−0.084	0.018	−4.948	−0.476

Variables entered: Audit score, HAMD score, HAMA score, PSC score, Liebowitz social anxiety scale, Emotional awareness, Emotional management, Social Emotional awareness, Relationship management, emotional work fatigue, physical work fatigue, mental work fatigue, suicidal ideation score, Number of kids, familial monthly income, marital status

Factor 1 = High emotional intelligence & low emotional work fatigue; Factor 2 = High physical and mental work fatigue & high stress; Factor 3 = Low self-esteem, high suicidal ideation and alcohol dependence.

Variables entered in the model: Factor 1, Factor 2, Factor 3, number of kids, familial monthly income, and marital status.

Variables entered in the model: cluster 1, cluster 2, cluster 3, number of kids, familial monthly income and marital status

^a^Reference = low familial monthly income

^b^cluster 1 = People with depersonalization (low emotional intelligence and high emotional work fatigue but low physical and mental work fatigue and low stress); cluster 2 = People with high wellbeing (high emotional intelligence and low emotional work fatigue, with high self-esteem, low suicidal ideation and low alcohol dependence); cluster 3 = People in distress (low self-esteem, high suicidal ideation and high alcohol dependence, with high physical and mental work fatigue and high stress)

## Discussion

Our study, the first of its kind in Lebanon, aimed at assessing risk factors associated with alexithymia among the general population. Our results showed that stress, mental and emotional work fatigue, alcohol dependence and anxiety were associated with more alexithymia, whereas being married was associated with less alexithymia.

Our population was divided into three clusters. Results of the present study were fairly expected for many reasons that make Lebanese people vulnerable to mental disorders: in 2003, a study has shown that nearly 50 % of the Lebanese population was confronted to traumatic events related to war [63]. The unstable political condition in Lebanon would consequently have an expected increase in the aforementioned percentage of the affected population. Lebanon had experienced a series of wars, local armed conflicts and terrorist attacks [35], in addition to the absence of clean water, 24-h electricity and problems with waste management [35, 64]. Add to this the high number of Syrian refugees that caused high unemployment rates [64] and xenophobic attitudes among Lebanese patients [65]. This is concurrent with the stigma of the public towards mental disorders [66], and the taboo associated with the search for a treatment for such disorders [67].

In this context, emotional disturbances (anxiety and depression) were shown to be associated with higher experienced traumas [68] and alexithymia [69]. Thus, recognizing and communicating feelings become crucial for the reduction of traumatic stress symptoms in persons experiencing a higher number of traumas [70].

Alexithymia prevalence was high in Lebanon compared to Finland [16], Germany [10] and Japan [71]. Our result, 20.8% (age range: 18–85 years), is probably higher because our study included a young aged group (in their 20s), scoring relatively high in TAS-20 scores [72].

Higher alcohol use disorder, revealed by a higher AUDIT score, was associated with more alexithymia. Our study corroborate the findings of a review article [73] since higher self-reports of alcohol consumption, stress and nicotine craving were associated with challenges identifying and describing feelings [73]. In addition, alcohol is consumed to alleviate tense conditions and improve interpersonal performance in individuals with alexithymia [74]. Alexithymic persons state consuming alcohol to feel more outgoing, friendly and confident, not to mention the easing effect of alcohol on the expression of their feelings [15]. Alcohol use is related to life discontent, and people would be more likely to drink to forget about their problems and be more likely to commit suicide [75, 76]. Suicidal ideation and attempts are related to psychiatric disorders including depression, anxiety, and substance use. It is assumed that these problems would be a mediator between alexithymia and suicidal ideation.

Our study results showed that anxiety was associated with an increase in alexithymia. This fact is in agreement with results of previous studies [77, 78], but not with those of Bach et al. [79] who, contrary to our results, did not find any significant association between alexithymia and various anxiety disorders. Few studies cited in a review [73] have included anxiety, depression or both as a covariate, highlighting that the association between alexithymia and alcohol is significant even after considering the effect of mood changes. Apparently, anxious individuals tend to limit their emotional experiences. This behavior could be explained as a protection strategy against the problems caused by somatic reactivity resulting from negative feelings [80].

Stress was also shown to be associated with alexithymia, similar to previous findings as well [81–83]. It seems that people with alexithymia use defensive mechanisms such as denial and repression of their emotions, while suppressing these emotions would lead to an intensity of negative emotions, anxiety, and depression [84].

Our findings showed that the burnout syndrome, expressed by a high emotional and mental work fatigue score, was associated with increased alexithymia, in line with results from previous studies [16, 85, 86]. The association between alexithymia and burnout is new but not that between emotional intelligence and burnout. Emotional intelligence, a notion solidly related to alexithymia, is explained as “*the ability to monitor one’s own and others’ feelings and emotions, to discriminate among them, and to use this information to guide one’s thinking and actions*” [87]. Some studies have shown that lower emotional intelligence is coupled to job-related burnout in human service work [88, 89]. In addition, emotional labor, i.e., customizing emotions when the job description asks that certain expressions should be shown to clientele, is a potential risk factor for emotional fatigue [90] and work-related exhaustion [91].

Perceived stress has been described to be related to higher level of burnout [92, 93]. It reflects the individual’s circumstances and relationships characterized by emotional exhaustion and impaired personal relationships usually related to professional life (workplace stressors) [94]. Consequently, a lack of adjustments to challenging situations will make these persons more susceptible to alexithymia. Thus, alexithymia may appear as a risk factor for burnout, particularly in human social work, which would constitute an interesting topic for prospective research projects.

Regarding sociodemographic characteristics and their relation to alexithymia, only a married status showed a lower alexithymia score. Several studies has pointed out the significant association between alexithymia and marital status [95–97]. Other studies has shown a link between being single or unmarried and difficulties to express emotions [98, 99]. These results may reflect the demographic and sociocultural principles that are found in Lebanon. This might be due to the close family ties and intergenerational solidarity, which is one of the main characteristics of Arabic countries where the secure style is predominant. In addition, intermediate and high socioeconomic statuses were associated with lower alexithymia, similar to the findings of Lane et al [95] The positive correlation between emotional consciousness and didactic realization goes along with the conceptualization of emotional awareness as a field of cognitive improvement that is highly affected by environmental determinants [95].

### Clinical implications

Multiple aspects might be associated with alexithymia that is, according to this study, common in the Lebanese population, and driven by stress, anxiety, alcohol use disorder and burnout. Thus, it is paramount to raise awareness about these factors and prevent alexithymia rather than treating it. Since the avoidance of negative emotions and stress is practically not possible, the person should know the way of regulating his/her emotions and deal with stressors, which may be essential to cope with emotions, whether positive or negative.

### Limitations

The results of this study cannot be extrapolated to the whole population since the majority were young (mean age: 30.30 years), had a university level of education and were single. The cross-sectional design of the study, cannot lead to conclusions as to whether alexithymia makes the population more prone to work-related burnout or anxiety or whether it is a secondary occurrence to these risk factors. Logically, the likelihood of alexithymia being a secondary phenomenon, a defense, consequential to extended mental stress and/or anxiety, cannot be ignored. The Arabic versions of the scales used have not been validated yet. Also, the retrospective and observational nature of the study result in a low level of evidence, and may increase the probability of information bias: overestimation of consequences for some known risk factors, problems in understanding question, and recall issues.

## Conclusion

Alexithymia appears to be influenced by many factors, including stress, anxiety, and burnout. To reduce its prevalence, it is important that health professionals educate the public about these factors. Further studies on a larger scale are needed to confirm our findings.

## Data Availability

All data generated or analyzed during this study are not publicly available to maintain the privacy of the individuals’ identities. The dataset supporting the conclusions is available upon request to the corresponding author.

## Abbreviations

3D-WFI: Three-Dimensional Work Fatigue Inventory
AUDIT: Alcohol Use Disorders Identification Test
C-SSRS: Columbia-Suicide Severity Rating Scale
HAM-A: Hamilton anxiety scale
HDRS: Hamilton depression rating scale
KMO: Kaiser–Meyer–Olkin
LSAS: Liebowitz Social Anxiety Scale
PSS: Perceived Stress Scale
RSES: Rosenberg self-esteem scale
TAS-20: Toronto Alexithymia Scale

## References

[1] Taylor GJ, Bagby RM (2000). An overview of the alexithymia construct.

[2] Parker JD, Shaughnessy PA, Wood LM, Majeski SA, Eastabrook JM (2005). Cross-cultural alexithymia: validity of the 20-item Toronto alexithymia scale in north American aboriginal populations. J Psychosom Res.

[3] Sifneos PE. Short-term psychotherapy and emotional crisis. Cambridge: Harvard University Press; 1972.

[4] Taylor Graeme, Bagby Michael, Parker James (1997). The development and regulation of affects. Disorders of Affect Regulation.

[5] Kaplan MJ, Dwivedi AK, Privitera MD, Isaacs K, Hughes C, Bowman M (2013). Comparisons of childhood trauma, alexithymia, and defensive styles in patients with psychogenic non-epileptic seizures vs. epilepsy: implications for the etiology of conversion disorder. J Psychosom Res.

[6] Ogrodniczuk JS, Piper WE, Joyce AS (2005). The negative effect of alexithymia on the outcome of group therapy for complicated grief: what role might the therapist play?. Compr Psychiatry.

[7] Faramarzi M, Azadfallah P, Book HE, Tabatabaei KR, Taheri H, Shokri-shirvani J (2013). A randomized controlled trial of brief psychoanalytic psychotherapy in patients with functional dyspepsia. Asian J Psychiatr.

[8] Faramarzi M, Shokri-Shirvani J, Kheirkhah F (2012). The role of psychiatric symptoms, alexithymia, and maladaptive defenses in patients with functional dyspepsia.

[9] Taylor G, Bagby R, Parker J (1997). Dissorders of affect regulation.

[10] Franz M, Popp K, Schaefer R (2008). Alexithymia in the German general population. Soc Psychiatry Psychiatr Epidemiol.

[11] Salminen JK, Saarijarvi S, Aarela E, Toikka T, Kauhanen J (1999). Prevalence of alexithymia and its association with sociodemographic variables in the general population of Finland. J Psychosom Res.

[12] Mattila AK, Salminen JK, Nummi T, Joukamaa M (2006). Age is strongly associated with alexithymia in the general population. J Psychosom Res.

[13] Pasini A, Delle Chiaie R, Seripa S, Ciani N (1992). Alexithymia as related to sex, age, and educational level: results of the Toronto alexithymia scale in 417 normal subjects. Compr Psychiatry.

[14] Nehra D, Sharma V, Mushtaq H, Sharma N, Sharma M, Nehra S (2011). Emotional intelligence and alexithymia in alcoholics. Behav Sci.

[15] Thorberg FA, Young RM, Sullivan KA (2011). Alexithymia in alcohol dependent patients is partially mediated by alcohol expectancy. Drug Alcohol Depend.

[16] Mattila AK, Ahola K, Honkonen T, Salminen JK, Huhtala H, Joukamaa M (2007). Alexithymia and occupational burnout are strongly associated in working population. J Psychosom Res.

[17] Taycan O, Taycan SE, Çelik C (2014). Relationship of burnout with personality, alexithymia, and coping behaviors among physicians in a semiurban and rural area in Turkey. Arch Environ Occup Health.

[18] Honkalampi K, Saarinen P, Hintikka J, Virtanen V, Viinamäki H (1999). Factors associated with alexithymia in patients suffering from depression. Psychother Psychosom.

[19] Kim JH, Lee SJ, Rim HD, Kim HW, Bae GY, Chang SM (2008). The relationship between alexithymia and general symptoms of patients with depressive disorders. Psychiatry Investig.

[20] Leweke F, Leichsenring F, Kruse J, Hermes S (2012). Is alexithymia associated with specific mental disorders. Psychopathology.

[21] Ogłodek EA, Szota AM, Just MJ, Araszkiewicz A, Szromek AR (2016). Sense of alexithymia in patients with anxiety disorders comorbid with recurrent urticaria. Neuropsychiatr Dis Treat.

[22] Scimeca G, Bruno A, Cava L, Pandolfo G, Muscatello MRA, Zoccali R (2014). The relationship between alexithymia, anxiety, depression, and internet addiction severity in a sample of Italian high school students. Sci World J.

[23] Ertekin E, Koyuncu A, Aslantaş Ertekýn B, Özyildirim I (2015). Alexithymia in social anxiety disorder: is there a specific relationship or is it a feature of comorbid major depression?. Anatolian J Psychiatry.

[24] Luminet O, Bagby RM, Taylor GJ (2001). An evaluation of the absolute and relative stability of alexithymia in patients with major depression. Psychother Psychosom.

[25] Cox BJ, Swinson RP, Shulman ID, Bourdeau D (1995). Alexithymia in panic disorder and social phobia. Compr Psychiatry.

[26] Taylor GJ, Bagby RM (2004). New trends in alexithymia research. Psychother Psychosom.

[27] Honkalampi K, Hintikka J, Tanskanen A, Lehtonen J, Viinamäki H (2000). Depression is strongly associated with alexithymia in the general population. J Psychosom Res.

[28] Marchesi C, Fontò S, Balista C, Cimmino C, Maggini C (2005). Relationship between alexithymia and panic disorder: a longitudinal study to answer an open question. Psychother Psychosom.

[29] Honkalampi K, Hintikka J, Laukkanen E, Viinamäki JLH (2001). Alexithymia and depression: a prospective study of patients with major depressive disorder. Psychosomatics.

[30] Yelsma P (1995). Self-esteem and alexithymia. Psychol Rep.

[31] Berastegui C, van Leeuwen N, Chabrol H (2012). Relationships between emotional intelligence, alexithymia and interpersonal delinquent behaviour in a sample of high-school students. Encephale.

[32] Parker JD, Taylor GJ, Bagby RM (2001). The relationship between emotional intelligence and alexithymia. Personal Individ Differ.

[33] Şahİn NH, Güler M, Basim HN (2009). The relationship between cognitive intelligence, emotional intelligence, coping and stress symptoms in the context of type a personality pattern. Turk J Psychiatry.

[34] Chen J, Xu T, Jing J, Chan RC (2011). Alexithymia and emotional regulation: a cluster analytical approach. BMC Psychiatry.

[35] Cherri Z, González PA, Delgado RC (2016). The Lebanese–Syrian crisis: impact of influx of Syrian refugees to an already weak state. Risk Manage Healthc Policy.

[36] Karam E, El Chammay R, Richa S, Naja W, Fayyad J, Ammar W (2016). Lebanon: mental health system reform and the Syrian crisis. BJPsych Int.

[37] Karam EG, Mneimneh ZN, Dimassi H (2008). Lifetime prevalence of mental disorders in Lebanon: first onset, treatment, and exposure to war. PLoS Med.

[38] Obeid S, Sacre H, Haddad C, et al. Factors associated with fear of intimacy among a representative sample of the Lebanese population: the role of depression, social phobia, self-esteem, intimate partner violence, attachment, and maladaptive schemas. Perspect Psychiatr Care. 2019. 10.1111/ppc.12438. 10.1111/ppc.12438 31549436

[39] Zakhour M, Haddad C, Salameh P, et al. Impact of the interaction between alexithymia and the adult attachment styles in participants with alcohol use disorder. Alcohol. 2019. 10.1016/j.alcohol.2019.08.007. 10.1016/j.alcohol.2019.08.007 31476366

[40] Lahoud N, Zakhour M, Haddad C (2019). Burnout and its relationships with alexithymia, stress, self-esteem, depression, alcohol use disorders, and emotional intelligence: results from a Lebanese cross-sectional study. J Nerv Ment Dis.

[41] Obeid S, Fares K, Haddad C, et al. Construction and validation of the Lebanese fear of relationship commitment scale among a representative sample of the Lebanese population. Perspect Psychiatr Care. 2019. 10.1111/ppc.12424. 10.1111/ppc.12424 31353477

[42] Haddad C, Zakhour M, Akel M (2019). Factors associated with body dissatisfaction among the Lebanese population. Eat Weight Disord.

[43] Khansa W, Haddad C, Hallit R, et al. Interaction between anxiety and depression on suicidal ideation, quality of life, and work productivity impairment: results from a representative sample of the Lebanese population. Perspect Psychiatr Care. 2019. 10.1111/ppc.12423. 10.1111/ppc.12423 31321788

[44] Obeid S, Haddad C, Akel M, Fares K, Salameh P, Hallit S (2019). Factors associated with the adults’ attachment styles in Lebanon: the role of alexithymia, depression, anxiety, stress, burnout, and emotional intelligence. Perspect Psychiatr Care.

[45] Saade S, Hallit S, Haddad C (2019). Factors associated with restrained eating and validation of the Arabic version of the restrained eating scale among an adult representative sample of the Lebanese population: a cross-sectional study. J Eat Disord.

[46] Haddad C, Hallit R, Akel M, et al. Validation of the Arabic version of the ORTO-15 questionnaire in a sample of the Lebanese population. Eat Weight Disord. 2019. 10.1007/s40519-019-00710-y. 10.1007/s40519-019-00710-y 31119588

[47] Haddad C, Obeid S, Akel M (2019). Correlates of orthorexia nervosa among a representative sample of the Lebanese population. Eat Weight Disord.

[48] Hamaideh SH (2018). Alexithymia among Jordanian university students: its prevalence and correlates with depression, anxiety, stress, and demographics. Perspect psychiatr Care.

[49] Bagby RM, Parker JD, Taylor GJ (1994). The twenty-item Toronto alexithymia scale--I. item selection and cross-validation of the factor structure. J Psychosom Res.

[50] Bagby RM, Taylor GJ, Parker JD (1994). The twenty-item Toronto alexithymia scale--II. Convergent, discriminant, and concurrent validity. J Psychosom Res.

[51] Thorberg FA, Young RM, Sullivan KA (2010). A confirmatory factor analysis of the Toronto alexithymia scale (TAS-20) in an alcohol-dependent sample. Psychiatry Res.

[52] Bohn MJ, Babor TF, Kranzler HR (1995). The alcohol use disorders identification test (AUDIT): validation of a screening instrument for use in medical settings. J Stud Alcohol.

[53] Rosenberg M. Society and the adolescent self-image. Princeton: Princeton university press; 1965.

[54] Obeid S., Abi Elias Hallit C., Haddad C., Hany Z., Hallit S. (2018). Validation of the Hamilton Depression Rating Scale (HDRS) and sociodemographic factors associated with Lebanese depressed patients. L'Encéphale.

[55] Hamilton M (1960). A rating scale for depression. J Neurol Neurosurg Psychiatry.

[56] Hamilton M (1959). The assessment of anxiety states by rating. Br J Med Psychol.

[57] Hallit Souheil, Haddad Chadia, Hallit Rabih, Akel Marwan, Obeid Sahar, Haddad Georges, Soufia Michel, Khansa Wael, Khoury Rony, Kheir Nelly, Abi Elias Hallit Christiane, Salameh Pascale (2019). Validation of the Hamilton Anxiety Rating Scale and State Trait Anxiety Inventory A and B in Arabic among the Lebanese population. Clinical Epidemiology and Global Health.

[58] Frone MR, Tidwell MO (2015). The meaning and measurement of work fatigue: development and evaluation of the three-dimensional work fatigue inventory (3D-WFI). J Occup Health Psychol.

[59] Nilsson ME, Suryawanshi S, Gassmann-Mayer C, Dubrava S, McSorley P, Jiang K (2013). Columbia–suicide severity rating scale scoring and data analysis guide. CSSRS Scoring Version.

[60] Liebowitz MR (1987). Social phobia. Mod Probl Pharmacopsychiatry.

[61] Rytwinski NK, Fresco DM, Heimberg RG (2009). Screening for social anxiety disorder with the self-report version of the Liebowitz social anxiety scale. Depress Anxiety.

[62] Mohapel P. The quick Emotional Intelligence Self-Assessment. San Diego City College MESA Program. Paul.mohapel@shaw.ca.2015.

[63] Karam EG, Mneimneh ZN, Karam AN (2006). 12-MONTH PREVALENCE AND TREATMENT OF MENTAL DISORDERS IN LEBANON: a National Epidemiologic Survey. Lancet.

[64] Turner BJ, Hollenbeak CS, Weiner M, Tang SS (2011). A retrospective cohort study of the potency of lipid-lowering therapy and race-gender differences in LDL cholesterol control. BMC Cardiovasc Disord.

[65] Obeid S, Haddad C, Salame W, Kheir N, Hallit S (2019). Xenophobic attitudes, behaviors and coping strategies among Lebanese people toward immigrants and refugees. Perspect Psychiatr Care.

[66] Abi Doumit C, Haddad C, Sacre H (2019). Knowledge, attitude and behaviors towards patients with mental illness: results from a national Lebanese study. PLoS One.

[67] Rayan Ahmad, Fawaz Mirna (2017). Cultural misconceptions and public stigma against mental illness among Lebanese university students. Perspectives in Psychiatric Care.

[68] Harder VS, Mutiso VN, Khasakhala LI, Burke HM, Ndetei DM (2012). Multiple traumas, postelection violence, and posttraumatic stress among impoverished Kenyan youth. J Trauma Stress.

[69] Haviland MG, Hendryx MS, Shaw DG, Henry JP (1994). Alexithymia in women and men hospitalized for psychoactive substance dependence. Compr Psychiatry.

[70] Park J, Jun JY, Lee YJ (2015). The association between alexithymia and posttraumatic stress symptoms following multiple exposures to traumatic events in north Korean refugees. J Psychosom Res.

[71] Shibata M, Ninomiya T, Jensen MP (2014). Alexithymia is associated with greater risk of chronic pain and negative affect and with lower life satisfaction in a general population: the Hisayama study. PLoS One.

[72] Moriguchi Y, Maeda M, Igarashi T (2007). Age and gender effect on alexithymia in large, Japanese community and clinical samples: a cross-validation study of the Toronto alexithymia scale (TAS-20). Biopsychosoc Med.

[73] Thorberg FA, Young RM, Sullivan KA, Lyvers M (2009). Alexithymia and alcohol use disorders: a critical review. Addict Behav.

[74] Finn PR, Martin J, Pihl RO (1987). Alexithymia in males at high genetic risk for alcoholism. Psychother Psychosom.

[75] Pompili M, Serafini G, Innamorati M (2010). Suicidal behavior and alcohol abuse. Int J Environ Res Public Health.

[76] Roy A, Linnoila M (1986). Alcoholism and suicide. Suicide Life Threat Behav.

[77] Marchesi C, Brusamonti E, Maggini C (2000). Are alexithymia, depression, and anxiety distinct constructs in affective disorders?. J Psychosom Res.

[78] Saarijärvi S, Salminen JK, Tamminen T, Äärelä E (1993). Alexithymia in psychiatric consultation-liaison patients. Gen Hosp Psychiatry.

[79] Bach M, Bach D, Bohmer F, Nutzinger DO (1994). Alexithymia and somatization: relationship to DSM-III-R diagnoses. J Psychosom Res.

[80] Besharat MA, Shahidi S (2011). What is the relationship between alexithymia and ego defense styles? A correlational study with Iranian students. Asian J Psychiatr.

[81] Nezhad SR, Rad MM, Farrokhi N, Viesy F, Ghahari S (2017). The relationship of alexithymia with depression, anxiety, stress, and fatigue among people under addiction treatment. Ann Trop Med Public Health.

[82] De Vente W, Kamphuis J, Emmelkamp P (2006). Alexithymia, risk factor or consequence of work-related stress?. Psychother Psychosom.

[83] Beshlideh K, Zakiei A, Sahraei Z, RajabiGilan N, Mohammadi O (2015). The simple, multiple, and canonical relationship between alexithymia and perceived stress with general health.

[84] Motan İ, Gençöz T (2007). The Relationship Between the Dimensions of Alexithymia and the Intensity of Depression and Anxiety. Turk J Psychiatry.

[85] Popa-Velea O, Diaconescu L, Mihăilescu A, Jidveian Popescu M, Macarie G (2017). Burnout and its relationships with alexithymia, stress, and social support among romanian medical students: a cross-sectional study. Int J Environ Res Public Health.

[86] Bodini B, Mandarelli G, Tomassini V (2008). Alexithymia in multiple sclerosis: relationship with fatigue and depression. Acta Neurol Scand.

[87] Salovey P, Mayer JD (1990). Emotional intelligence. Imagin Cogn Pers.

[88] Durán A, Extremera N, Rey L (2004). Self-reported emotional intelligence, burnout and engagement among staff in services for people with intellectual disabilities. Psychol Rep.

[89] Gerits L, Derksen JJ, Verbruggen AB, Katzko M (2005). Emotional intelligence profiles of nurses caring for people with severe behaviour problems. Personal Individ Differ.

[90] Totterdell P, Holman D (2003). Emotion regulation in customer service roles: testing a model of emotional labor. J Occup Health Psychol.

[91] Brotheridge CM, Grandey AA (2002). Emotional labor and burnout: comparing two perspectives of “people work”. J Vocat Behav.

[92] Dahlin ME, Runeson B (2007). Burnout and psychiatric morbidity among medical students entering clinical training: a three year prospective questionnaire and interview-based study. BMC Med Educ.

[93] Pöhlmann K, Jonas I, Ruf S, Harzer W (2005). Stress, burnout and health in the clinical period of dental education. Eur J Dent Educ.

[94] Maslach C, Schaufeli WB, Leiter MP (2001). Job burnout. Annu Rev Psychol.

[95] Lane RD, Sechrest L, Riedel R (1998). Sociodemographic correlates of alexithymia. Compr Psychiatry.

[96] Epözdemir H (2012). The effect of alexithymic characteristics of married couples on their marital adjustment. J Fam Psychother.

[97] Hazan C, Shaver P (1987). Romantic love conceptualized as an attachment process. J Pers Soc Psychol.

[98] Kauhanen J, Kaplan G, Julkunen J, Wilson T, Salonen J (1993). Social factors in alexithymia. Compr Psychiatry.

[99] Kokkonen P, Karvonen JT, Veijola J (2001). Prevalence and sociodemographic correlates of alexithymia in a population sample of young adults. Compr Psychiatry.

